# Effects of functional training with blood occlusion on the irisin, follistatin, and myostatin myokines in elderly men

**DOI:** 10.1186/s11556-022-00303-2

**Published:** 2022-09-24

**Authors:** Fatemeh Pazokian, Sadegh Amani-Shalamzari, Hamid Rajabi

**Affiliations:** Department of Exercise Physiology, Faculty of Physical Education and Sports Science, Kharazmi University, South Mofatteh Ave, Tehran, Iran

**Keywords:** Hypertrophy, Occlusion training, Weight training, Muscle strength

## Abstract

**Background:**

This study aimed to determine the efficacy of functional training with and without blood flow restriction (BFR) on muscle hypertrophy indices and strength in older men.

**Methods:**

Thirty older adults (67.7 ± 5.8 years) were randomly assigned to three groups: functional training (FT), functional training with BFR (FTBFR), and control (C). Participants in experimental groups were trained in three sessions per week for six weeks. They performed 11 whole body exercises, in 2–4 sets of 10 repetitions. FTBFR group wore pneumatic cuffs on their extremities that began with 50% of estimated arterial occlusion pressure which increased by 10% every two weeks. Blood samples were obtained, and static strength tests were evaluated at baseline and after the training program. A One-Way Analysis of Covariance was used to interpret the data.

**Results:**

A significant increase in follistatin levels (*p* = 0.002) and reduction in myostatin levels (*p* = 0.001) were observed in FT and FTBFR groups; there was a considerable increase in the F:M ratio in both training groups (*p* = 0.001), whereas it decreased in C group. These changes were accompanied by significant improvements in handgrip (*p* = 0.001) and shoulder girdle (*p* = 0.001) strength in both experimental groups, especially in the FTBFR group. However, the levels of irisin were not statistically changed following interventions (*p* = 0.561).

**Conclusion:**

The findings showed that FT was effective in increasing circulating biomarkers involved in hypertrophy in older adults while adding BFR to FT had a slight increase in these biomarkers but had a tremendous increase in muscle strength.

## Background

Aging is considered a natural process in human life rather than a pathological issue. However, this natural process is often accompanied by undesirable structural changes and a decline in physiological functions [1]. Muscle atrophy and a reduction in force-generating capacity arising from aging lead to diminished functional capacity and quality of life [2]. The mechanisms responsible for this decline in functional capacity with aging have not been fully elucidated. However, researchers have reported that age-related reduction of muscle mass and contractile capacity is manifested by changes in certain levels of serum biomarkers [3–5].

Myokines are peptides or proteins released from skeletal muscle and are involved in multiple physiological processes, including metabolism and hypertrophy in the autocrine and paracrine manner [6]. Myostatin, as a muscle atrophy index, increases in the older population [7]. It binds to the activin type II receptor and suppresses muscle growth signaling pathways, protein synthesis, satellite cell activation, and negatively regulates myogenic differentiation and myofiber hypertrophy [8, 9]. In contrast, follistatin as a member of the transforming growth factor-β family, blocking myostatin [4] and activating satellite cells [10], leading to hypertrophy and preventing age-associated muscle wastes. Research has shown that serum follistatin levels decrease by aging [11]. Since myostatin and follistatin bind to the same receptor, the follistatin to myostatin (F:M) ratio is associated with muscle atrophy or hypertrophy [12]. In addition, the circulatory level of irisin is acknowledged to positively correlate with muscle mass and strength [13–15], and its declined serum levels have been reported in older adults [16]; hence, irisin is also recognized as a circulating biomarker for sarcopenia [5]. Therefore, interventions such as exercise training that mitigate muscle loss may be associated with changes in the circulatory level of irisin and F:M ratio.

Functional training (FT), a popular form of resistance training, includes movements similar to daily life [17] seems an ideal workout routine for the older adult population. FT contains several sets of resistance exercises performed with body mass, elastic bands, or free weights on a stable or unstable surface [18]. According to a study both FT and traditional training were equally beneficial for improving strength components in older women [19]. Also, Liu et al. (2014), in the review article, reported the beneficial effects of FT on the daily activities of older adults [20]. Interestingly, these desirable adaptations have been linked with increases in serum follistatin levels and decreases myostatin mRNA expression in skeletal muscle [21, 22], and increases in circulating irisin levels in the older population [23]. Although FT effectively improves health indicators, it lacks a high metabolic load [24, 25]. It was reported a combination of the proper mechanical and metabolic loads is needed to prevent a loss of strength and muscle mass caused by aging [24]. A practical method for increasing the metabolic load of resistance training is blood flow restriction (BFR). In addition, individuals with chronic diseases and the older adult population who may not have the capacity to perform high-intensity training could use BFR simultaneously with exercise training to impose both mechanical and metabolic loads on the skeletal muscles [26].

Training with BFR is a new model of training that increases exercise intensity and provides similar or greater adaptations than high-intensity training. The primary physiological mechanism of BFR is tissue ischemia, which results in the accumulation of byproducts and cellular swelling, leading to the release of growth factors [27]. It was shown that training with BFR substantially increases blood lactate, growth hormone, and insulin-like growth factor-1 concentrations [28, 29], which ultimately up-regulate protein synthesis, leading to maintaining or improving muscle mass. Hence, we assumed that using BFR during FT generates further physiological adaptations in the older adult population. Although adaptations to FT in older adults have been previously investigated, the effect of FT with BFR on the sarcopenia index and functional ability of older adult individuals warrants further investigation. To our knowledge, no study has investigated the effects of these interventions in elderly individuals. Therefore, the primary aim of this study was to examine the impact of FT with BFR on fitness levels and the secondary aim was to determine serum levels of myostatin, follistatin, and irisin in older adults. We hypothesized that performing FT with BFR improves the performance capacity and F:M ratio and irisin levels greater than FT alone.

## Methods

### Study design

**Fig. 1:**
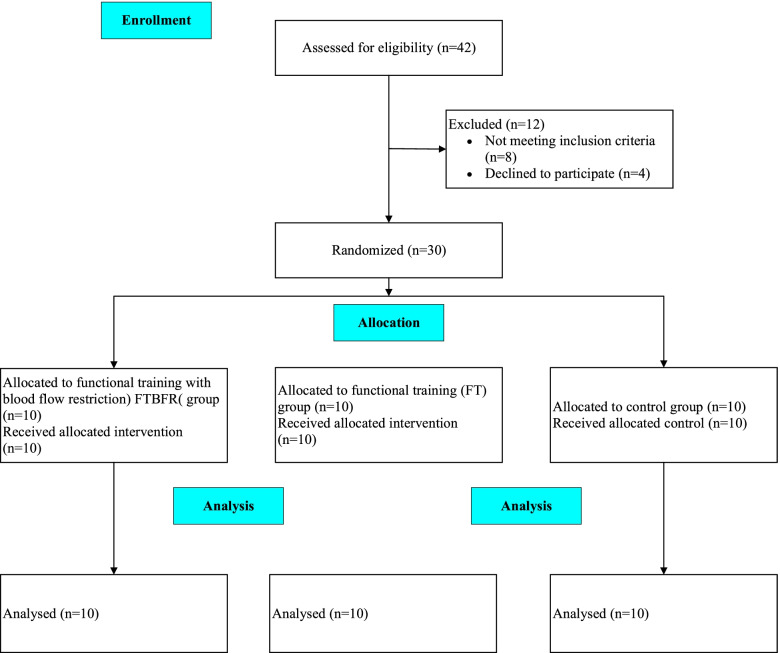
Schematic flow chart of the study timeline

In this randomized trial, the performance capacity and circulatory levels of myostatin, follistatin, and irisin were measured following a 6-week functional training program with and without BFR. One week before the intervention, participants attended a familiarization session. In this session, they performed a battery of performance tests and reached a competency level to perform the tests correctly. Randomization was completed before the study by a third person who was not on the research team; eligible participants were assigned into three groups equally (*n* = 10), including the functional training (FT) group, functional training with blood flow restriction (FTBFR) group, and the control (C) group. Physical characteristics and pre-intervention performance indicators, including handgrip and shoulder girdle strength, were assessed 72 h, and blood samples were collected 48 h, before and after the intervention period by an expert who was not the author of this study. A flow diagram of the study is shown in Fig. 1.

### Participants

Men over the age of 60 from a retirement center volunteered to participate in this trial. Inclusion criteria included no history of cardiac disease, hypertension, diabetes mellitus, neuromuscular disorder, or metabolic illnesses. Participants who were involved in organized training more than once a week were also excluded. Participants did not have orthopedic conditions and were able to do their daily activities and pre-tests without limitation. To estimate the number of participants needed in the study, a sample size calculation was performed using G*Power Software version 3.1.9.6 (Düsseldorf, Germany) (Faul et al., 2007) for a one-way ANCOVA, using a rejection criterion of 0.05 and 0.85 (1-β) power, and large effect (f = 0.65). The power calculation indicated that a minimum of 10 participants was required to be able to find such an effect. Thirty older men (age: 67.7 ± 5.8 years; body mass: 72.4 ± 12.1 kg; body mass index: 25.8 ± 3.1 kg/m^2^) who met these criteria were included in this study. The block randomization way (size 6) was applied, to assign participants to each group. All procedures performed in this study were under the Helsinki statement regarding human research. The ethics committees for the Sport Sciences Research Institute of Iran approved this study by approval number: IR.SSRI.REC.1397.299.

### Test procedures

#### Anthropometry

A standard stadiometer (Seca 213, Germany) is used to measure the height. A calibrated digital scale (Seca 769, Germany) is used to measure the body mass after stomach defecation and after 8 to 10 h of fasting. Body fat percentage was obtained by the body composition device (InBody S10, Biospace Company Limited, Seoul, South Korea).

#### Handgrip strength

Handgrip strength was assessed by a hand dynamometer (HFEH11WBAA, handeful) to estimate static strength. Firstly, the width of the dynamometer’s handle had been adjusted to each subject’s hand size, and then measurements had been accomplished while participants were sitting in a straight position by holding their arms unsupported and parallel to the body. Participants were asked to exert maximal force and were given verbal encouragement during the trials. Each person performed three attempts by the dominant hand with 1.5 min of recovery time between measurements, and eventually, the highest value was recorded.

#### Shoulder girdle strength

One repetition maximum (1-RM) for shoulder press was estimated following the national strength and conditioning association guidelines for the assessment of 1RM dynamic strength. The assessor verbally encouraged the participants during the test to achieve the maximum number of repetitions.

#### Blood sampling and analysis

The venous blood samples were obtained by a laboratory specialist 48 h before and after the 6-week intervention while subjects were under 8 to 10 h of overnight fasting. To evaluate the study variables, the samples were collected into 5 cc Ethylenediaminetetraacetic acid tubes. After that, blood samples were centrifuged at 8000 rpm at 4 °C for 10 min, and the collected serum was stored at − 20 ◦C for analysis. The irisin (CSB-EQ027943HU, Cusabio, USA), myostatin (CSB-E11300h, Cusabio, USA), and follistatin (CSB-E-13516 h, Cusabio, USA) concentrations were determined using Enzyme-Linked Immunosorbent Assays. The sensitivity of the irisin kit was 0.78 ng/mL, and inter-assay and intra-assay were CV % < 10% and CV % < 8%, respectively. The sensitivity of myostatin was 0.312 ng/mL and inter-assay and intra-assay were CV % < 15% and CV % < 15%. The sensitivity of the follistatin kit was 3.12 ng/mL, and inter-assay and intra-assay were CV % < 10% and CV % < 8%, respectively.

#### Blood flow restriction protocol

BFR was applied by wearing 5 cm pneumatic cuffs (Ghamat pooyan, Tehran, Iran) on the proximal portion of the arms and legs. The cuffs were inflated by manual pumps during each set and were deflated during the rest times between sets. Arterial occlusion pressure (AOP) was estimated using the formula (1) for upper and (2) for lower body parts [30].

1 $$\mathrm{Upper body arterial occlusion }(\mathrm{mmHg})= (1.461 \times \mathrm{ arm circumference}) +( 0.514 \times \mathrm{ systolic blood pressure}) + (0.339 \times \mathrm{ diastolic blood pressure}) + 17.236$$

2 $$\mathrm{Lower body arterial occlusion }(\mathrm{mmHg})=(5.893 \times \mathrm{ thigh circumference}) + (0.912 \times \mathrm{ systolic blood pressure}) + (0.734 \times \mathrm{ diastolic blood pressure}) - 220.046$$

The cuff pressure in training sessions was set to 50% of the calculated AOP (∼210 − 250 mmHg for the lower body and 105 − 130 mmHg for the upper body) in weeks 1 and 2; then, the pressure increased 10% every two weeks (60% in weeks 3–4, and 70% in weeks 5–6). In addition, the rate of perceived exertion was recorded to monitor training intensity.

### Training intervention

Functional training programs were performed three times per week on non-consecutive days for six weeks. Each training session began by walking on a treadmill and stair climber with their rhythm for 10 min as a warm-up. Eleven functional exercises were selected and designed in the form of circuit training as follows: 1) dumbbell fly on a swiss ball; 2) wall squat with a swiss ball; 3) triceps extension while lying on a swiss ball; 4) forward lunge on a Bosu ball; 5) shoulder press while standing on a Bosu ball; 6) medicine ball squat throw; 7) standing biceps curl with dumbbells on a Bosu ball; 8) Leg curl with a powerband while lying on a Bosu ball; 9) seated row with power bands on Bosu ball; 10) super crunches with a medicine ball; 11) medicine ball hyperextension on the swiss ball. Details of the training protocol including, set, repetition, and intensity of exercises, are shown in Table 1. The intensity of exercises was calculated based on %1-RM for stations 1 to 5 and 7, the mass of a medicine ball for stations 6, 10, and 11; and the color of the bands for stations 8 and 9. (Blue, Black, and Red bands have low, medium and high resistance, respectively). Certified exercise instructors supervised all exercise sessions. In the meantime, participants in the C group sustained their lifestyles.

**Table 1:** Training protocol during the 6 weeks of intervention

Variables	Cuff pressure (AOP%)	Sets (N)	Repetitions (N)	Rest between set (min)	Intensity		
					*Dumbbells (%1RM)*	*Medicine ball (kg)*	*Powerband (color)*
Week 1&2	50	2	10	1	25%	3	Blue
Week 3&4	60	3	10	1	30%	4	Black
Week 5&6	70	4	10	1	35%	5	Red

### Statistical analyses

The Statistical Package of Social Sciences (SPSS, IBM, version19) was used to perform statistical analysis. Data were presented as the mean ± standard deviation. First, normal distribution of data using Shapiro–Wilk's test; homogeneity of variance using box plot, and homogeneity of regression slopes were confirmed, and then a One-Way Analysis of Covariance (ANCOVA) was used to compare post-intervention values between groups, while the pre-test values were included as covariates. If there were significant differences between groups, Bonferroni post-hoc tests were performed to determine the differences between groups. Bivariate Pearson’s product-moment correlation coefficient (r) was calculated to assess the magnitude and direction of the linear relationships between the changes in circulatory blood markers and performance (post-test data). Paired sample t-tests were performed to analyze intragroup changes in response to the intervention. Effect sizes were also estimated to examine the magnitude of differences while controlling for the influence of the sample size [31].

## Result

There were no significant differences between groups at the start of the intervention in all physical and blood biomarkers. The physical characteristics of participants were presented in Table 2. Training intervention could not statistically change the body mass, BMI, and body fat (*p* > 0.05).

**Table 2:** Anthropometric characteristics of participants (Mean ± SD)

		FTBFR	FT	C
Age (years)		67.6 ± 5.1	66.3 ± 4.6	69.3 ± 7.4
Height (cm)		166.2 ± 5.6	165.3 ± 9.6	170.2 ± 9.7
Body mass (kg)	Pre	69.8 ± 11.5	71.4 ± 5.5	75.9 ± 10.7
	post	68.6 ± 11.3	70.9 ± 5.2	76.0 ± 10.7
BMI (kg/m^2^)	Pre	25.5 ± 3.4	25.8 ± 1.7	26.2 ± 2.9
	post	24.9 ± 3.7	25.7 ± 1.7	26.3 ± 2.8
Body Fat (%)	Pre	23.1 ± 5.4	23.3 ± 2.5	22.9 ± 5.3
	Post	22.8 ± 5.7	22.7 ± 2.4	23.4 ± 5.5

*FTBFR* Functional training with blood flow restriction, *FT* Functional training, *C* control, *cm* Centimeter, *kg* Kilogram, *BMI* Body mass index, *kg/m*^2^ Kilogram per square meter, *%* percentage

**Fig. 2:**
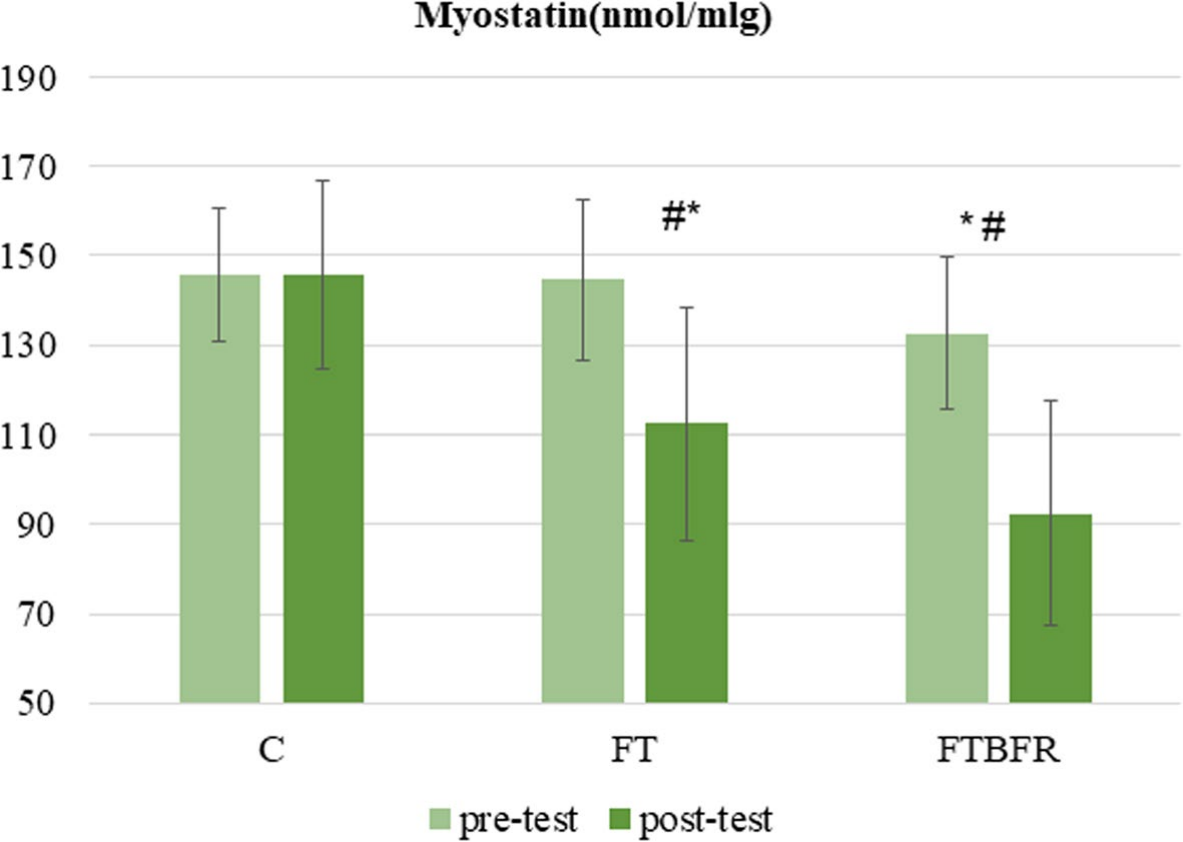
Myostatin concentration before and after the intervention. FTBFR: Functional training with blood flow restriction; FT: Functional training; C: control; *significantly different from pre-intervention; # significantly different from the control group

The ANCOVA results indicated a significant difference between groups at myostatin levels (F = 10.9, *p* = 0.001, η^2^ = 0.46). The post-hoc analysis showed myostatin levels were significantly lower in FT and FTBFR groups compared to the C group (Fig. 2). However, the circulatory levels of myostatin in the FT group were not significantly different from FTBFR (*p* > 0.05). Paired t-tests were performed to assess intra-group variations and the results indicated that the circulatory levels of myostatin decreased considerably by 30.7% in the FTBFR group (*t* = 6.7, *p* = 0.001), and 21.7% in the FT group (*t* = 3.9, *p* = 0.004) and without change (0.01%) in the C group (*t* = 0.01, *p* = 0.99).

**Fig. 3:**
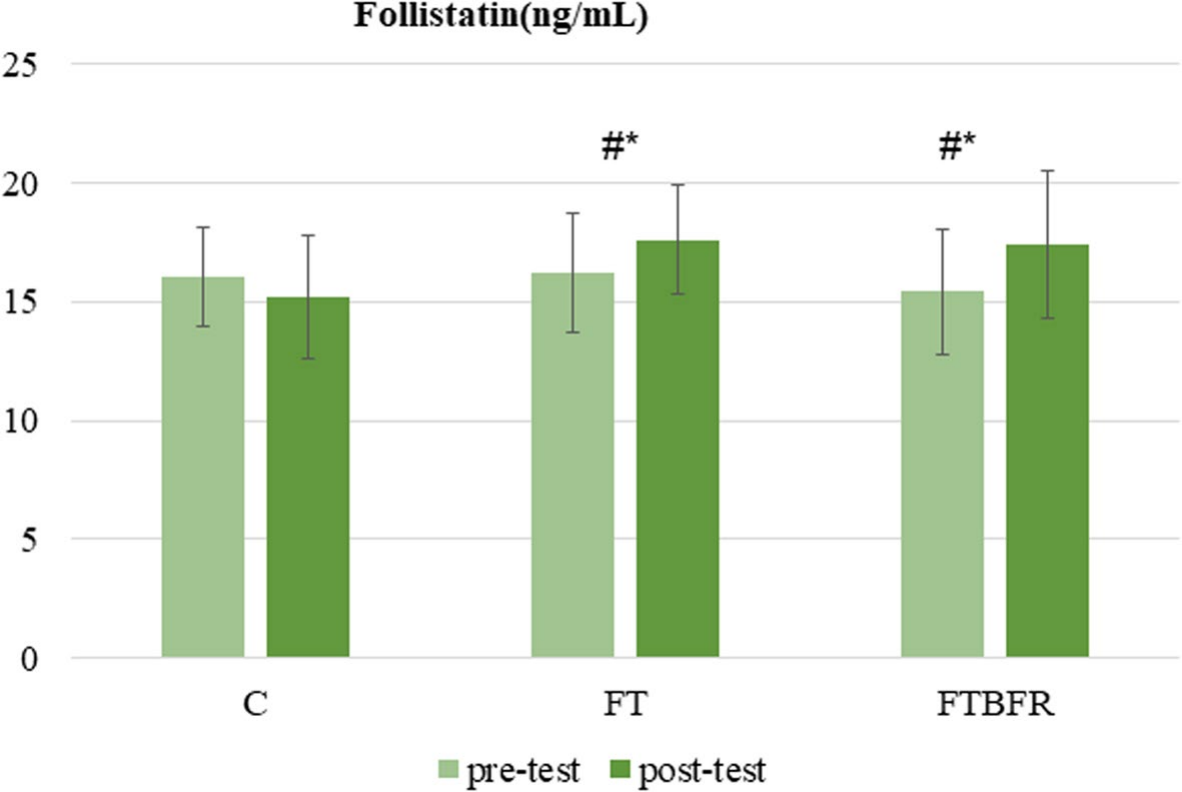
Follistatin concentration before and after the intervention. FTBFR: Functional training with blood flow restriction; FT: Functional training; C: control; *Significantly different from pre-intervention; # Significantly different from the control group

Follistatin levels after the intervention differed significantly between groups (F = 8.1, *p* = 0.002, *η*^2^ = 0.38) (Fig. 3). The post-hoc test demonstrated that the circulatory levels of follistatin increased significantly in the FT and FTBFR groups compared to the C group, and there was no significant difference between the FTBFR and FT groups. Moreover, according to the findings of the paired sample t-tests, the follistatin level decreased by -5.0% in the C group (*t* = 1.8, *p* = 0.108) and increased by 9.7% in the FT group (*t* = 3.2, *p* = 0.010), and decreased by 13.7% in the FTBFR group (*t* = 3.3, *p* = 0.010).

**Fig. 4:**
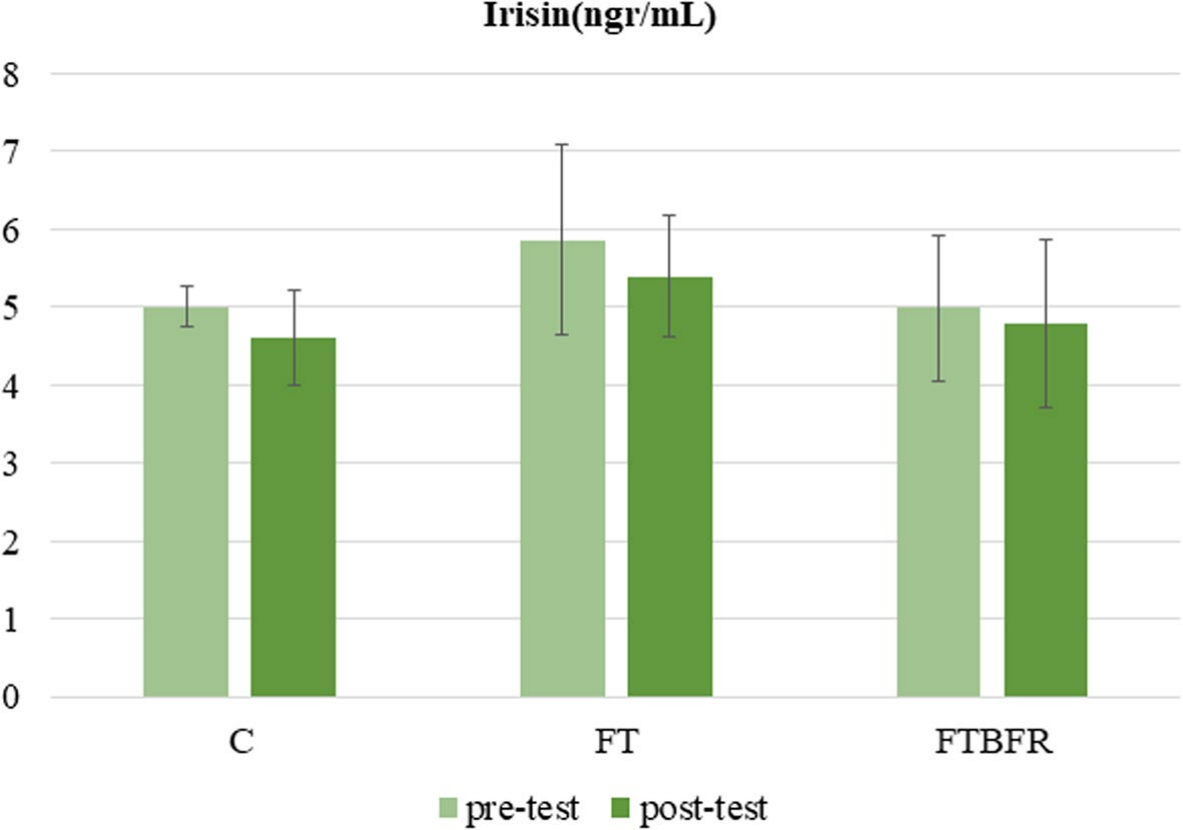
Irisin concentration before and after the intervention. FTBFR: Functional training with blood flow restriction; FT: Functional training; C: control

After the intervention, there was no statistically significant difference in the levels of irisin between the groups (F=0.6, *P*=0.561, η2 =0.04) (Fig. 4). Although the circulatory irisin level decreased -7.6% in C group (t=1.8, *p*=0.094), -5.5% in the FT group (t=1.4, *p*=0.183) and - 0 0.5 1 1.5 2 2.5 C FT FTBFR Follistatin(ng/mL) pre-test post-test #* #* 17 3.3 in the FTBFR group (t=0.7, *p*=0.512), but there were not any significant changes intragroups as shown the result of paired t-test.

**Fig. 5:**
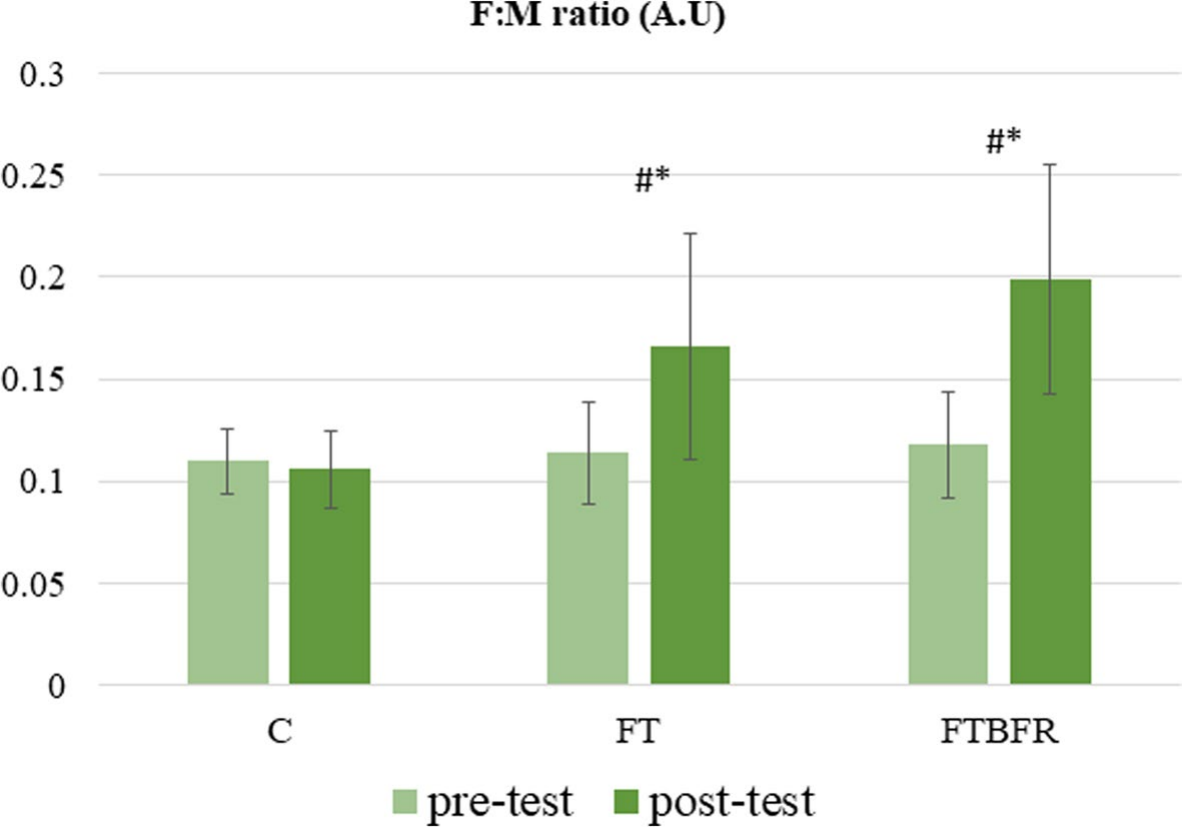
Follistatin to Myostatin (F:M) ratio before and after the intervention. FTBFR: Functional training with blood flow restriction; FT: Functional training; C: control; *significantly different from pre-intervention; # significantly different from the control group

Moreover, there was a significant difference in F:M ratio between groups (F = 11.7, *P* = 0.001, *η*^2^ = 0.47); the post-hoc test also showed the difference between the C group and the two experimental groups (Fig. 5). The F:M ratio decreased by -4.5% in the C group (*t* = 1.3, *p* = 0.236) and increased by 47.1% in the FT group (*t* = 3.7, *p* = 0.005), and increased by 73.5% in the FTBFR group (*t* = 5.1, *p* = 0.001).

**Fig. 6:**
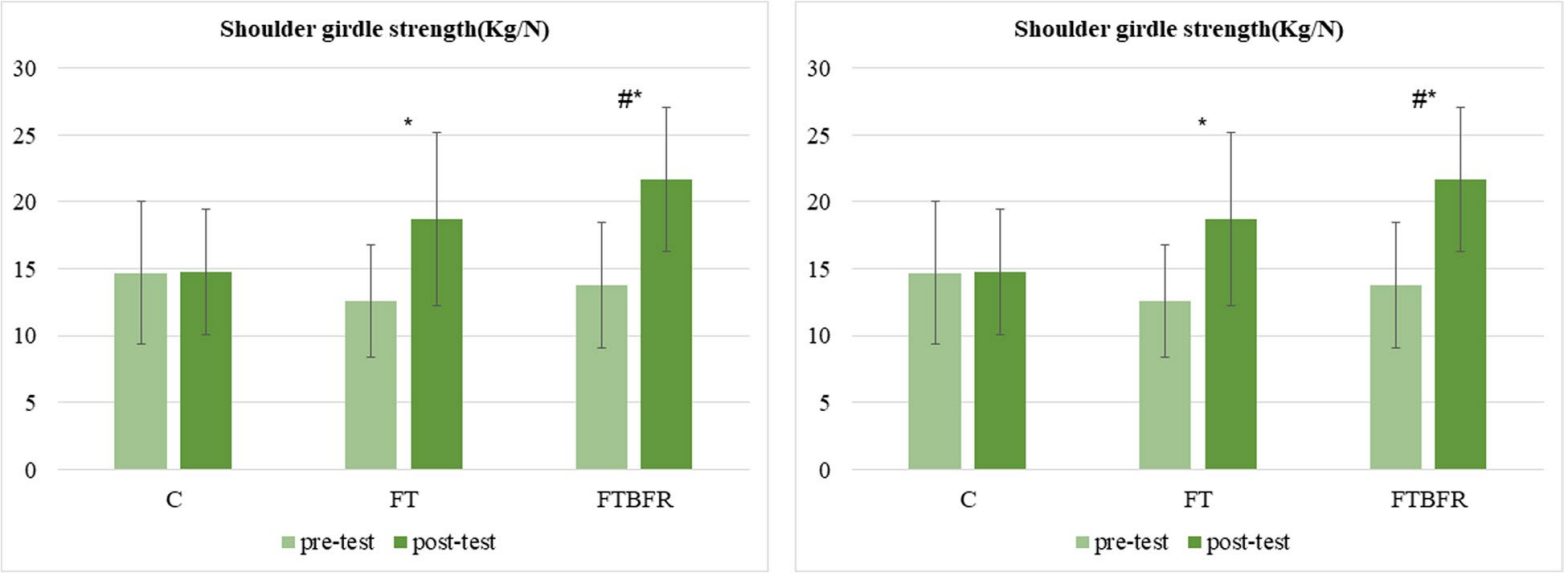
**a** Handgrip strength level (Kg/N) and (**b**) Shoulder girdle strength level (Kg/N) before and after the intervention. FTBFR: Functional training with blood flow restriction; FT: Functional training; C: control; *significantly different from pre-intervention # significantly different from the control group

Following the intervention, substantial improvements were observed in handgrip (F = 9.8, *P* = 0.001, η^2^ = 0.43) and shoulder girdle strength (F = 16.9, *P* = 0.001, η^2^ = 0.57) in both experimental groups, especially in the FTBFR group (*p* > 0.05) (Fig. 6 a, b). The post-hoc test showed that the differences were between the experimental groups with the C group. There was no significant difference between the FT and FTBFR groups.

The average rating of perceived exertion (RPE) during the intervention period was approximately 16–18 for the FTBFR group and 14–16 for the FT group. It seems that participants in the FTBFR group experienced greater training exertion compared to participants in the FT group (*p* ≤ 0.01). Despite this greater training exertion in the FTBFR group, there was no significant difference in performance outcomes between the FT and FTBFR groups.

## Discussion

The primary aim of this study was to measure and compare the efficacy of functional training with and without BFR on fitness level and serum concentrations of myostatin, follistatin, and irisin in older men. The results showed that in the FT and FTBFR groups, in comparison to the C group, there was a notable decrease in the serum levels of myostatin and a large increase in the circulatory levels of follistatin. However, there was little difference between the experimental groups. Accordingly, the F:M ratio in the FT and FTBFR groups showed significant changes in relation to the C group. The findings also manifested that irisin circulatory levels did not change significantly following the interventions. Moreover, participants in both FT and FTBFR groups demonstrated a considerable improvement in handgrip and shoulder girdle strength. The rate of increase in handgrip strength was significantly different from the C group only in the FTBFR group. At last, the findings of this study indicated FT could alter circulating biomarkers involved in hypertrophy in older adults, and adding BFR to FT has a slight increase in blood biomarkers and a tremendous increase in strength over a short period.

The findings showed that FT led to a substantial increase in body mass and shoulder girdle and handgrip strength compared to the C group, while the addition of BFR only slightly increased these indices over the short period of 6 weeks. Our results are supported by previous research that revealed FT is more efficient than traditional training, which is often performed in uni-axial and stable machines to improve power, mobility rate, and quality of life in the older population [20, 32]. It has been shown that the increase in muscle strength is primarily due to neuromuscular adaptations, increased activity of agonist muscles [33] and reduced activity of antagonist muscles [33, 34]. Moreover, training with BFR provides a greater stimulus for hypertrophy [35] by demanding more oxygen, accumulating metabolites and recruiting fast-twitch muscle fibers that are leading to increased strength and power [35, 36] as well as preventing atrophy and sarcopenia [37], which are mainly influenced on the fast-twitch fibers. Increased strength is associated with molecular changes. In this regard, fife et al.(2018) demonstrated a negative relation between the circulating plasma myostatin and follistatin and muscle function in older adults [38]. Our findings supported it by showing that the change percentage in strength was consistent with the change percentage in the F:M ratio in the two experimental groups. In addition, previous studies reported that resistance training leads to decreased myostatin levels and increased follistatin plasma levels [39, 40].

The F:M ratio is now recognized as a crucial indicator of body composition and muscle strength resulting from resistance training [12, 41]. Our findings showed an increase in this ratio in the two experimental groups that resulted from the observed decreased myostatin levels and increased follistatin levels. Previous studies have already approved these results and shown exercise training reduces and increases circulatory myostatin and follistatin levels respectively [42–44]. In this regard, Negaresh et al.(2017) showed that eight weeks of resistance training decreased myostatin levels and increased follistatin levels [44], so the F:M ratio increases, leading to the anabolic conditions to maintain and improve muscle mass. On the other hand, adding BFR to FT further decrease myostatin by increasing metabolic load. Laurentino et al. (2012) previously supported this finding and reported that low-intensity resistance training with BFR is as effective in reducing myostatin levels as heavy resistance training [39]. It seems that applying mechanical and metabolic loads to the muscle cell by increasing the insulin-like growth factor-1 and follistatin inhibits the activity of the FOX1 pathway, which consequently reduces the expression of activin serine/threonine ӀӀ receptors, leading to ultimately reducing the expression and secretion of myostatin [45]. In addition, the decreased myostatin could be attributed to the self-regulatory theory of myostatin. In this theory myostatin in a negative feedback loop through the smad-7 signal pathway, reduces the expression of the myostatin gene in muscle cells [46]. On the other hand, the release of muscle growth factors, which suppress the myostatin gene expression correlates with the exercise intensity and muscle mass involved in the activity [47]. Thus, the further increase in the F:M ratio in the FTBFR group can be attributed to the greater recruitment of fast-twitch fibers in this group [48]. Therefore, by activating intracellular signaling pathways, FT reduces the production of myostatin and the addition of BFR by exerting a metabolic load exacerbates these changes.

There were no significant changes in the circulatory irisin level following the intervention. Consistent with our results, studies reported no significant changes in serum irisin levels after eight weeks of resistance training [49] and 21 weeks of combined strength-endurance training [50]. In addition, although an increase in plasma irisin level has been reported after short bouts of intensive exercise, no significant changes were observed in the long-term resistance training protocol [51, 52]. In contrast, some researchers reported that irisin levels increase after long-term resistance training intervention [23, 53]. These studies lasted 12 weeks, and the large muscles were trained at a moderate intensity, which differed from our findings; thus, it appears that longer periods of resistance training involving large muscles with moderate to high loads are required for tangible effects on irisin concentration. However, it remains unclear whether resistance or strength training could affect circulating irisin levels.

We acknowledge that there were some limitations in this research. First, due to the unavailability of Doppler as the standard method of determining the amount of arterial occlusion, it was estimated from equations as already developed [30]. Second, the small sample size may affect observed results; however, this effect was mitigated using a magnitude-based assessment. Finally, it was difficult to measure the exercise intensity; however, Borg's 10-point mental assessment was used.

## Conclusion

Overall, FT over six weeks led to a considerable improvement in handgrip and shoulder girdle strength, which was associated with decreased serum myostatin and increased serum follistatin, hence the F:M ratio increased. Adding BFR to FT only slightly modified the measured cytokines in the blood, although it had a relatively more pronounced effect on performance variables. Therefore, individuals older than 65 years are recommended to perform FT to maintain muscle mass, and applying BFR during these exercises has more pronounced effects.

## Data Availability

The dataset used and analyzed during the current study is available from the corresponding author on reasonable request.

## Abbreviations

BFR: Blood flow restriction
FT: Functional training
FTBFR: Functional training with blood flow restriction
C: Control
F:M: Follistatin to Myostatin
1-RM: One repetition maximum
AOP: Arterial occlusion pressure
SPSS: Statistical Package of Social Sciences
ANCOVA: Analysis of Covariance
RPE: Rating of perceived exertion
