# Correction: Effects of functional training with blood occlusion on the irisin, follistatin, and myostatin myokines in elderly men

**DOI:** 10.1186/s11556-022-00308-x

**Published:** 2022-11-02

**Authors:** Fatemeh Pazokian, Sadegh Amani-Shalamzari, Hamid Rajabi

**Affiliations:** Department of Exercise Physiology, Faculty of Physical Education and Sports Science, Kharazmi University, South Mofatteh Ave, Tehran, Iran

**Correction: Eur Rev Aging Phys Act 19, 22 (2022)**

10.1186/s11556-022-00303-2

Following publication of the original article [1], the authors identified errors in Figs. 2 and 3 (units were written incorrectly). The correct figures are given below.

**Fig. 2:**
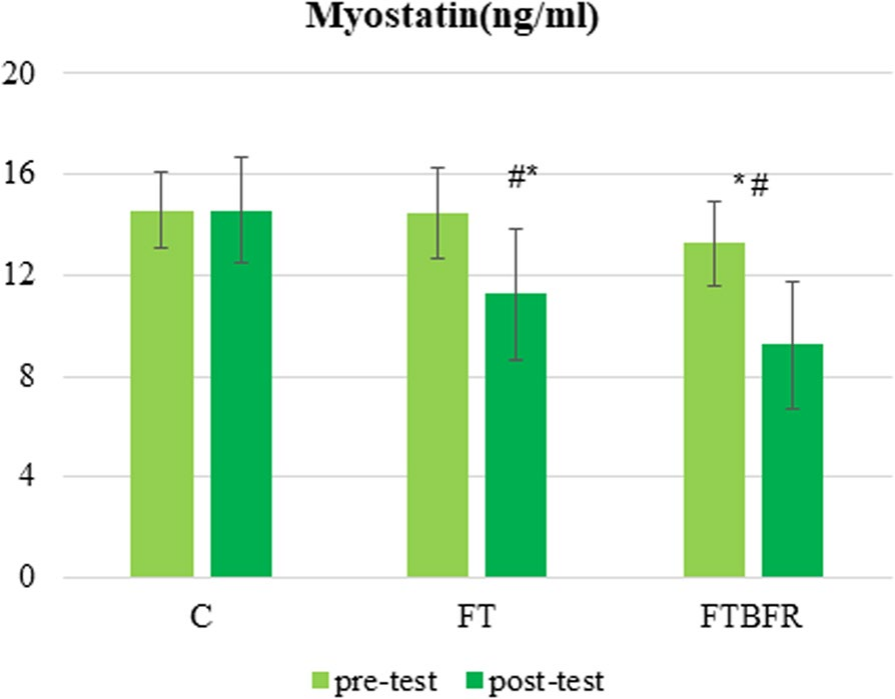
Myostatin concentration before and after the intervention. FTBFR: Functional training with blood flow restriction; FT: Functional training; C: control; *significantly different from pre-intervention; # significantly different from the control group

**Fig. 3:**
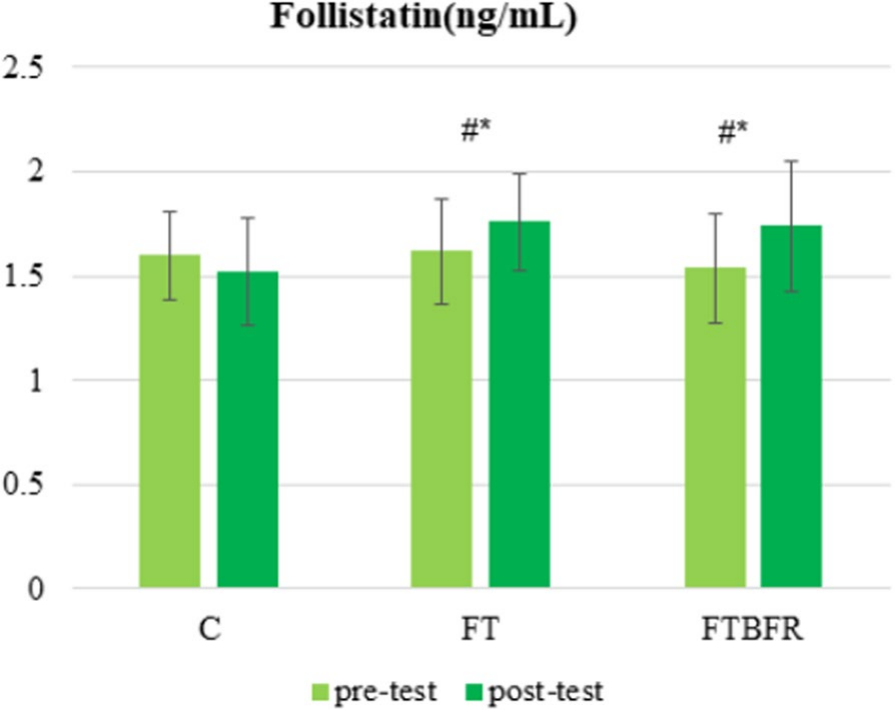
Follistatin concentration before and after the intervention. FTBFR: Functional training with blood flow restriction; FT: Functional training; C: control; *Significantly different from pre-intervention; # Significantly different from the control group

## References

[1] Pazokian F, Amani-Shalamzari S, Rajabi H (2022). Effects of functional training with blood occlusion on the irisin, follistatin, and myostatin myokines in elderly men. Eur Rev Aging Phys Act.

